# Mouse Models of Influenza Infection with Circulating Strains to Test Seasonal Vaccine Efficacy

**DOI:** 10.3389/fimmu.2018.00126

**Published:** 2018-01-31

**Authors:** Helen T. Groves, Jacqueline U. McDonald, Pinky Langat, Ekaterina Kinnear, Paul Kellam, John McCauley, Joanna Ellis, Catherine Thompson, Ruth Elderfield, Lauren Parker, Wendy Barclay, John S. Tregoning

**Affiliations:** ^1^Mucosal Infection and Immunity Group, Section of Virology, Department of Medicine, St Mary’s Campus, Imperial College London, London, United Kingdom; ^2^The Crick Institute, London, United Kingdom; ^3^Respiratory Virus Unit, Public Health England, London, United Kingdom; ^4^Molecular Virology, Section of Virology, Department of Medicine, St Mary’s Campus, Imperial College London, London, United Kingdom

**Keywords:** Influenza Vaccines, mouse models, Infection, Antibodies, Viral, vaccine drift

## Abstract

Influenza virus infection is a significant cause of morbidity and mortality worldwide. The surface antigens of influenza virus change over time blunting both naturally acquired and vaccine induced adaptive immune protection. Viral antigenic drift is a major contributing factor to both the spread and disease burden of influenza. The aim of this study was to develop better infection models using clinically relevant, influenza strains to test vaccine induced protection. CB6F1 mice were infected with a range of influenza viruses and disease, inflammation, cell influx, and viral load were characterized after infection. Infection with circulating H1N1 and representative influenza B viruses induced a dose-dependent disease response; however, a recent seasonal H3N2 virus did not cause any disease in mice, even at high titers. Viral infection led to recoverable virus, detectable both by plaque assay and RNA quantification after infection, and increased upper airway inflammation on day 7 after infection comprised largely of CD8 T cells. Having established seasonal infection models, mice were immunized with seasonal inactivated vaccine and responses were compared to matched and mismatched challenge strains. While the H1N1 subtype strain recommended for vaccine use has remained constant in the seven seasons between 2010 and 2016, the circulating strain of H1N1 influenza (2009 pandemic subtype) has drifted both genetically and antigenically since 2009. To investigate the effect of this observed drift on vaccine induced protection, mice were immunized with antigens from A/California/7/2009 (H1N1) and challenged with H1N1 subtype viruses recovered from 2009, 2010, or 2015. Vaccination with A/California/7/2009 antigens protected against infection with either the 2009 or 2010 strains, but was less effective against the 2015 strain. This observed reduction in protection suggests that mouse models of influenza virus vaccination and infection can be used as an additional tool to predict vaccine efficacy against drift strains.

## Introduction

Influenza infection is a significant cause of morbidity and mortality worldwide; the WHO estimates that there are 3–5 million severe influenza cases every year, causing 250,000–500,000 deaths globally (1). There is also a considerable economic burden from influenza epidemics, which cost the European economy approximately €6–€14 billion and the US economy $87.1billion annually (2, 3). There are currently several different vaccines available including trivalent or quadrivalent inactivated vaccines and live attenuated vaccines. Seasonal influenza vaccination is considered the most effective intervention strategy for reducing the burden of influenza disease (4, 5). However, influenza vaccines have highly variable rates of efficacy, ranging from 10% in 2004–2005 (6) to 60% in 2010–2011 (7). The main cause of vaccine failure is mismatch between the vaccine and circulating strains. The cause of these mismatches is change in the circulating strains, either through antigenic drift (small mutations in hemagglutinin sequence) or antigenic shift (major replacements of circulating virus).

Due to the changing nature of the circulating influenza virus strain, vaccine strain selection mismatches can and do occur (8). In the autumn of 2014, increased rates of influenza activity were observed in the United States and this was attributed to poor vaccine effectiveness as a result of a mismatch between the H3 component of the current influenza vaccine and circulating strains (8). The overall effectiveness of the 2014–2015 influenza vaccine for preventing medically attended laboratory confirmed influenza virus was 23% (9). Early studies of influenza infections during the 2014/2015 season found that 100% of lab confirmed influenza A infections were A (H3N2) and of those 67% were antigenically drifted from A/Texas/50/2012, the reference strain used for the 2014/2015 vaccine in the northern hemisphere (9). A similar report from Canada found that 91% of the isolates were found to be genetically and antigenically distinct from the A/Texas/50/2012 vaccine strain (10). The same time period saw the emergence of a new lineage of H3N2 viruses (3C.2a and 3C.3a), which showed poor reactivity with antisera raised against A/Texas/50/2012 leading to its replacement with A/Switzerland/9715293/2013 in the next vaccine season (11).

Part of the decision process about which strains should be used for vaccines is hemagglutination inhibition (HI) using ferret sera, complemented with virus neutralization data. However, mice are widely used in the preclinical development and evaluation of potential vaccines and antiviral compounds and have the potential to inform decisions. In this paper, we develop models of influenza infection in CB6F1 mice and evaluate the effect of vaccination on disease outcome after infection with matched and mismatched strains of virus.

## Results

### Recent Clinical Isolates of H1N1 Subtype and Influenza B, but not H3N2 Cause Disease in Mice

**Figure 1:**
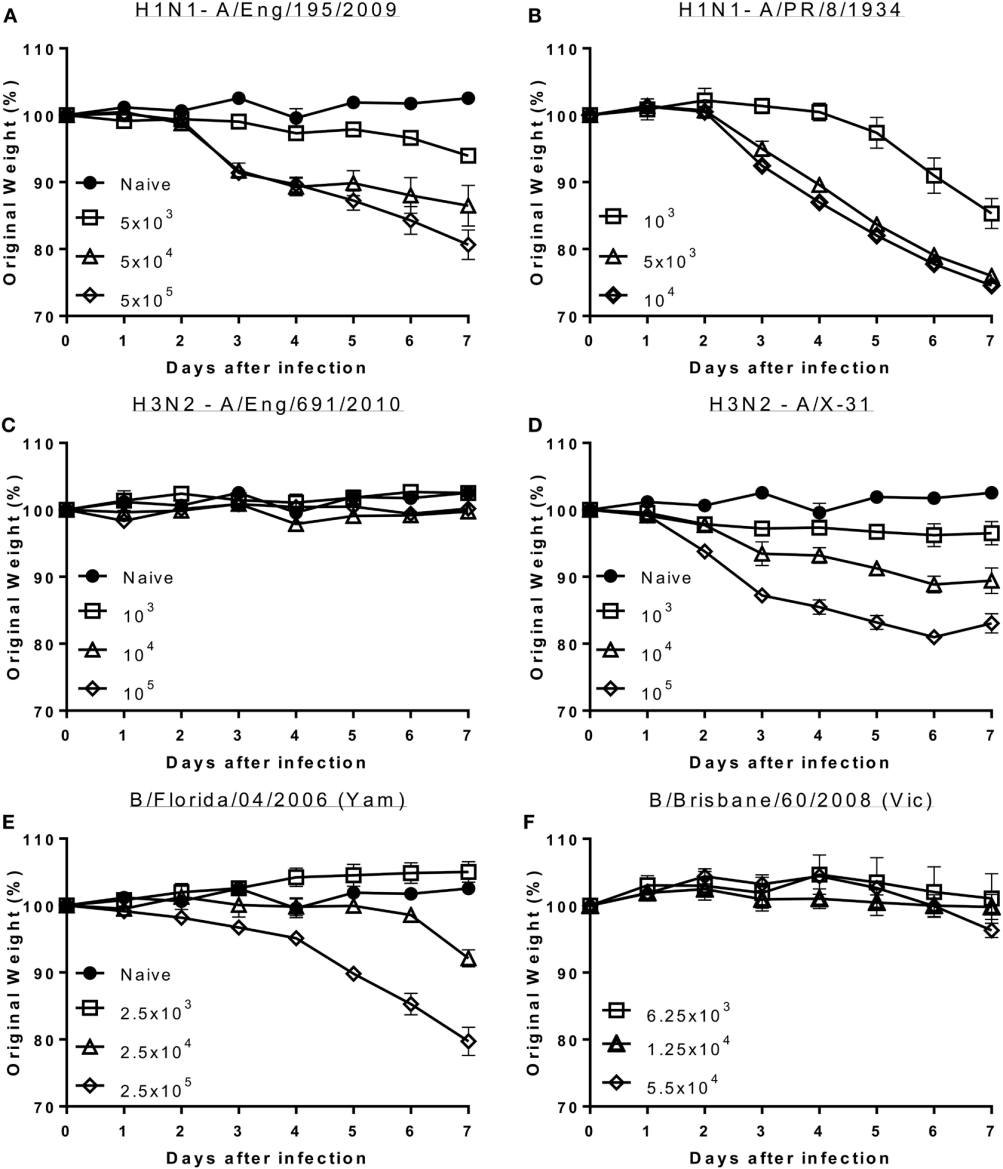
Seasonal influenza H1 and B but not seasonal H3 cause disease in mice. Mice were infected with increasing doses of different influenza viruses intranasally in 100 μl volumes. Weight loss was measured after infection with A/England/195/2009 **(A)**, A/Puerto Rico/8/1934 **(B)**, A/England/691/2010 **(C)**, A/X-31 **(D)**, B/Florida/04/06 (Yamagata) **(E)** or B/Brisbane/60/2008 (Victoria) **(F)**. Points represent mean of *n* ≥ 4 animals ± SEM.

Mice were infected with escalating doses of viruses reflective of recent circulating influenza viral strains and or widely used laboratory strains. For H1N1, mice were infected with influenza A/England/195/2009 (12) (clinical isolate: Figure 1A) or PR8 (Lab strain: Figure 1B). Animals infected with both the seasonal and laboratory strains of H1N1 lost weight proportionally to the infectious dose of virus. Comparing the response by dose of plaque forming units would suggest PR8 causes more disease per PFU used, but there may be limitations in using PFU for comparisons. For H3N2, mice were infected with A/England/691/2010 (Clinical isolate: Figure 1C) or A/X31 (Lab strain), which consists of HA and NA molecules from A/Hong Kong/1/68 (H3N2) on a PR8 background (Figure 1D). While animals infected with the laboratory H3N2 strain, X31, lost weight after infection, mice infected with the current seasonal H3N2 virus (A/England/691/2010) did not lose weight at the doses used. To test responses to influenza B, mice were infected with virus isolates that are close to current circulating strains, B/Florida/04/06 representing the Yamagata lineage (Figure 1E) and B/Brisbane/60/2008 (Figure 1F) representing the Victoria lineage. Infection with the Yamagata but not the Victoria lineage influenza B led to weight loss, but a larger dose of virus may be required for the Victoria lineage virus.

### Infectious and Immunological Characterization of Influenza Infection in Mice

**Figure 2:**
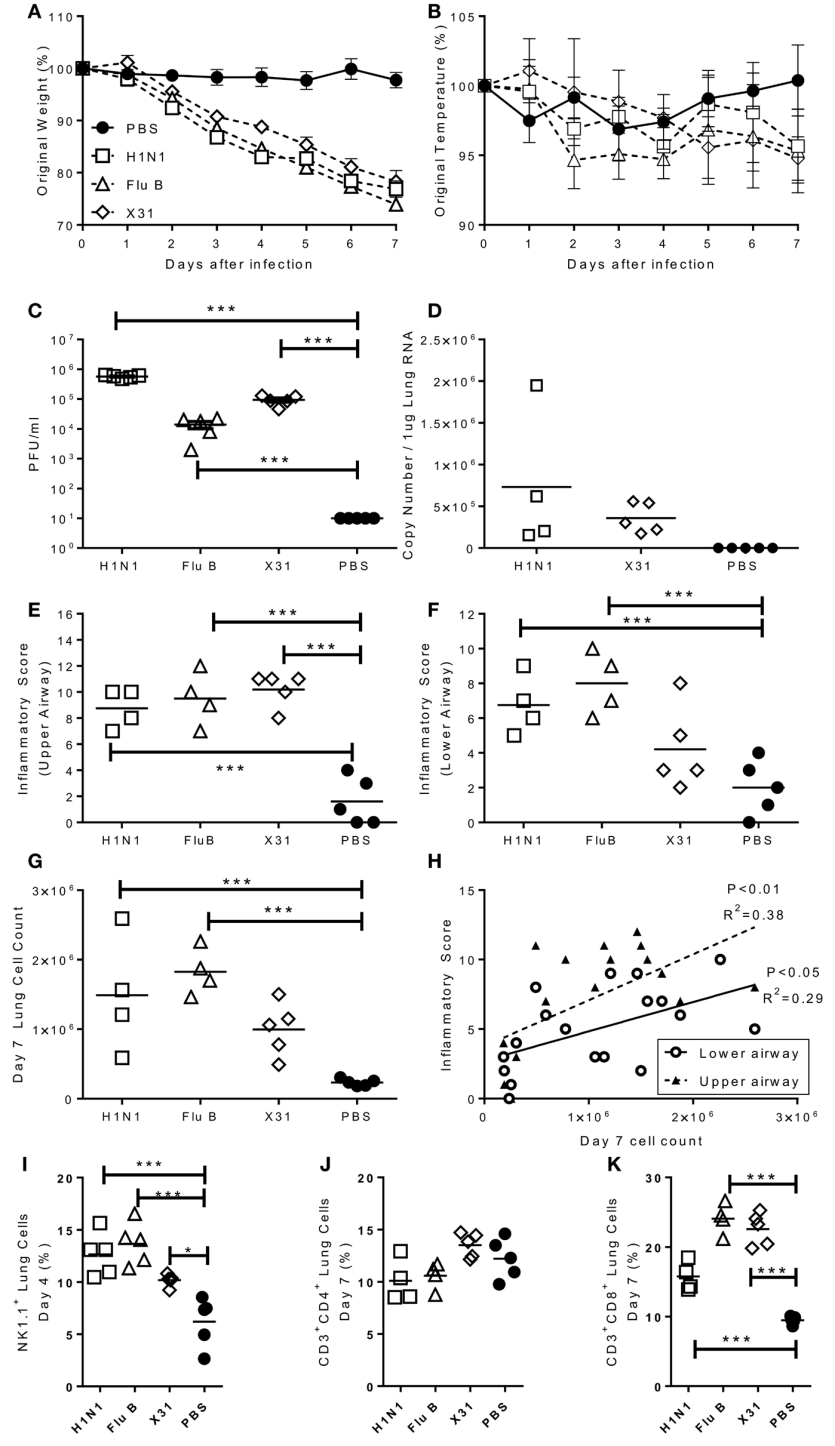
Characterization of pathogenic response to seasonal influenza infections. Mice were infected with H1N1, Flu B, or X31. Weight **(A)** and temperature **(B)** were measured daily after infection. Viral load was measured by plaque assay **(C)** or rt-PCR **(D)** on day 4 after infection. Inflammation in the upper **(E)** and lower **(F)** airways were measured on day 7 after infection. Cell numbers in the lung was assessed at day 4 were counted **(G)** and compared to inflammation score **(H)**. NK **(I)**, CD4 **(J)**, and CD8 **(K)** cells in lungs assessed by flow cytometry. Points represent means of *n* ≥ 4 animals ± SEM **(A,B)** or individual animals **(C–K)**.

Having observed that infection with some strains of influenza virus caused signs of disease, we wished to confirm that these viruses were able to replicate in mouse lungs and wanted to investigate the histological and immunological correlations of disease. Mice were challenged intranasally with representative H1N1 (Eng/195), Flu B (Flo/04), and H3N2 (A/X-31) strains and monitored over 7 days. A control group of mice were given sterile PBS intranasally. All influenza challenged mice lost significant amounts of weight compared to the control group (Figure 2A). Temperature was also measured, but no significant differences were observed (Figure 2B). Lung viral load was assessed *via* plaque assay (all groups) or influenza A M gene RNA qPCR (H1N1, X31, and control) (Figures 2C,D). Virus was detected in the lungs *via* plaque assay on day 4 for all infected mice (Figure 2C). Viral RNA was quantified for the influenza A infected groups and was detected on day 4 for both H1N1 and A/X-31 (Figure 2D). At day 7, virus and viral RNA was only detected in the H1N1 infected mice (data not shown).

Lung inflammation was investigated as a measure of disease pathology. There were no significant differences in lung inflammation at day 4 after challenge in either the upper and lower airways (data not shown). However, 7 days after challenge there was significantly more inflammation in both the upper (Figure 2E) and lower (Figure 2F) airways of infected animals. This lower airway inflammation was reflected by an increased cell recovery on day 7 after infection (Figure 2G). Airway inflammation correlated with lung cell counts (Figure 2H). The composition of the lung cellular infiltrate was assessed by flow cytometry. Four days after influenza challenge, there was a significant increase in the percentage of NK cells in infected mice compared to PBS controls (Figure 2I). The infiltrate at day 7 was predominantly made up of CD8 T cells, with no differences in the number of CD4 T cells (Figure 2J) but significantly higher levels of CD8 T cells in the lungs of infected mice than controls (Figure 2K).

### Mice Are Protected against Homologous Challenge Infection after Inactivated or Live Attenuated Vaccine

**Figure 3:**
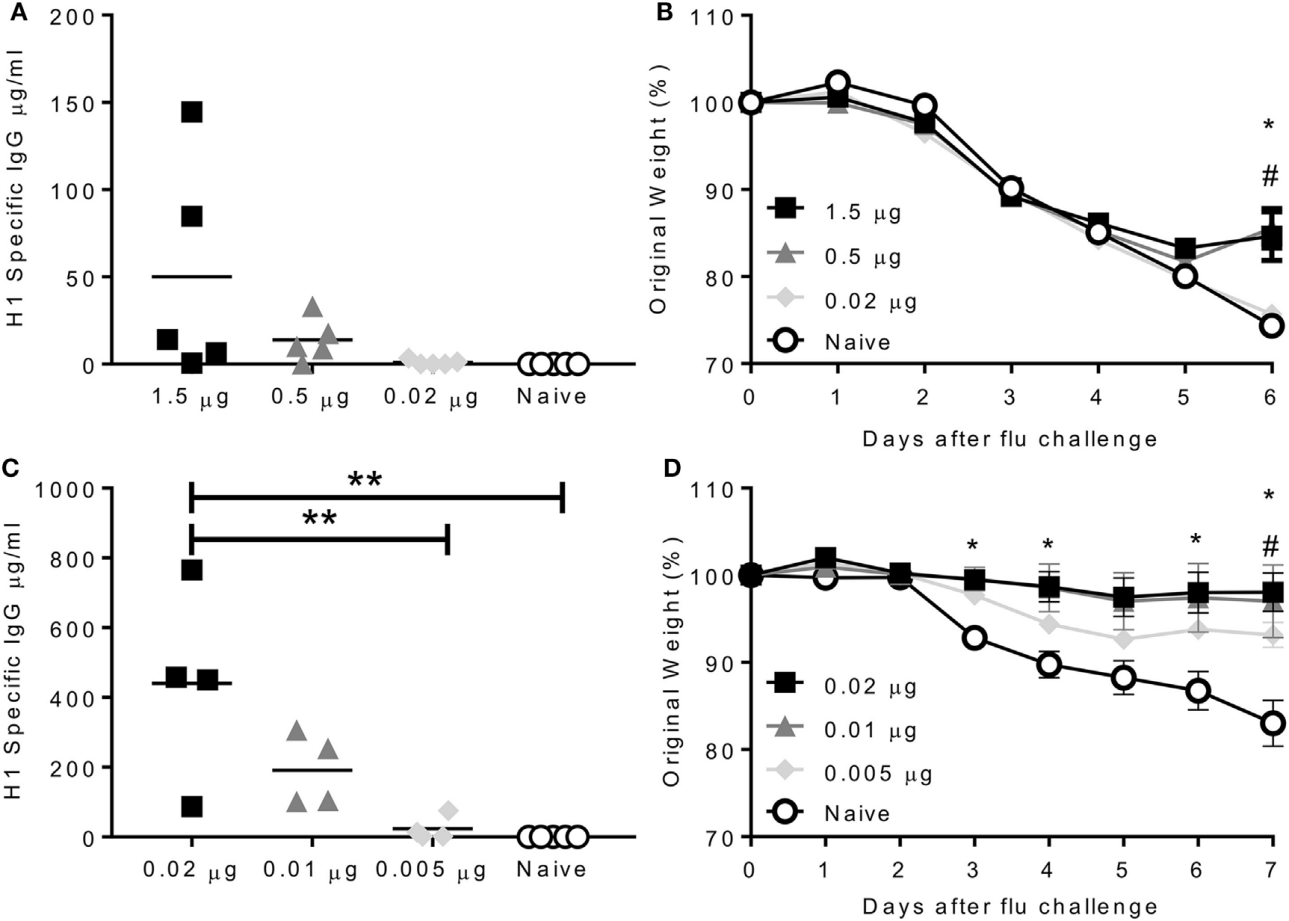
Immunization protects against homologous challenge. CB6F1 mice were immunized once with varying doses of H1N1 antigens. Antibody was measured prior to challenge **(A)** with A/California/7/2009 (H1N1pdm09), weight loss measured daily **(B)**. Mice were immunized twice with varying doses of H1N1 antigens. Antibody was measured prior to challenge **(C)** with A/California/7/2009 (H1N1pdm09), weight loss measured daily **(D)**. Points represent individual animals **(A,C)** or means of *n* ≥ 4 animals ± SEM **(B,D)**. Panel **(B)** *p < 0.05 between 1.5 µg and naïve group and #*p* < 0.05 between 1.5 µg and 0.02 µg group. Panel **(C)** **p < 0.01.

Having developed infectious challenge models, we wished to determine the efficacy of seasonal influenza vaccines in mice. Mice were intramuscularly immunized with purified surface antigens from A/California/7/2009, which was the (H1N1)pdm09 strain used in the trivalent vaccine from 2010 to 2016. The aim of the study was to find the lowest protective dose of vaccine, mice were given increasing doses from 0.02 to 1.5 µg A/California/7/2009 influenza hemagglutinin (as part of a mixture of viral surface antigens), for reference the human vaccine dose is 15 µg. The antibody response was proportional to the immunization dose, with most in the 1.5 µg immunized group (Figure 3A). Mice received a single dose of vaccine and were challenged with 2.5 × 10^5^ PFU A/California/7/2009 H1N1 4 weeks later. Mice immunized with 1.5 or 0.5 µg lost up to 15% body weight peaking day 5 after infection. These mice were partially protected compared to the naïve animals, losing significantly less weight than naïve animals on day 6 after infection (Figure 3B). Mice immunized once with 0.02 µg did not produce antibodies and were not protected against challenge. To test whether repeat immunization affected the dose required, mice were immunized with 0.02, 0.01, or 0.005 µg (20, 10, or 5 ng) of A/California/7/2009 H1N1 hemagglutinin on days 0 and 21. Antibody responses were significantly greater in mice immunized with 0.02 µg than 0.005 µg or the naïve animals (Figure 3C). Mice were protected against infection when mice were vaccinated twice in a prime boost regime with a dose of 0.02 or 0.01 µg and partial protection was seen after immunization with 0.005 µg (Figure 3D). From these studies, we observe that immunization with a very low dose of protein can protect mice against homologous influenza challenge.

### Influenza Drift Reduces the Efficacy of the Inactivated Vaccine Antigen

The biggest recent change in vaccine strains occurred with the emergence of the H1N1 pandemic strain in 2009. Because the strain recommendation preceded the emergence of the virus in 2009, the 2009–2010 vaccine did not contain the (H1N1)pdm09 like strain. However, from 2010 to 2016, the H1N1 subtype strain included in the virus was A/California/7/2009 (H1N1pdm09). By comparison, in the seven seasons since the emergence of the strain of H1N1 influenza (2009 pandemic strain) to the winter of 2017, the H3N2 component was changed four times (Table 1). In the same time period, the B component has changed between representative Yamagata and Victoria lineage reference strains in trivalent vaccines (13); after 2012 quadrivalent vaccines with two B strains were recommended. We wished to determine the genetic and antigenic drift of the (H1N1)pdm09 viral strains since its emergence in 2009.

**Table 1:** Recommended vaccine strains (Northern Hemisphere) 2010–2017.

Season	H1N1	H3N2	B	Additional B strain for QIV
2009–2010	A/Brisbane/59/2007	A/Brisbane/10/2007	B/Brisbane/60/2008 (Vic)	N/A
2010–2011	A/California/7/2009	A/Perth/16/2009	B/Brisbane/60/2008 (Vic)	N/A
2011–2012	A/California/7/2009	A/Perth/16/2009	B/Brisbane/60/2008 (Vic)	N/A
2012–2013	A/California/7/2009	A/Victoria/361/2011	B/Wisconsin/1/2010 (Yam)	B/Brisbane/60/2008 (Vic)
2013–2014	A/California/7/2009	A/Victoria/361/2011	B/Massachusetts/2/2012 (Yam)	B/Brisbane/60/2008 (Vic)
2014–2015	A/California/7/2009	A/Texas/50/2012	B/Massachusetts/2/2012 (Yam)	B/Brisbane/60/2008 (Vic)
2015–2016	A/California/7/2009	A/Switzerland/9715293/2013	B/Phuket/3073/2013 (Yam)	B/Brisbane/60/2008 (Vic)
2016–2017	A/California/7/2009	A/Hong Kong/4801/2014	B/Brisbane/60/2008 (Vic)	B/Phuket/3073/2013 (Yam)
2017–2018	A/Michigan/45/2015	A/Hong Kong/4801/2014	B/Brisbane/60/2008 (Vic)	B/Phuket/3073/2013 (Yam)

**Figure 4:**
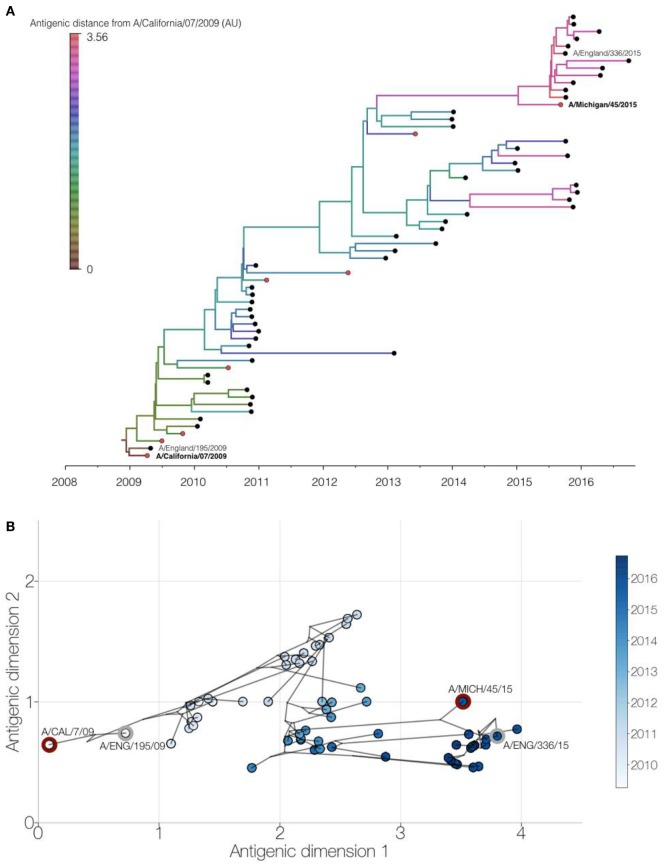
Genetic and antigenic drift of H1N1pdm09 strains between 2009 and 2016. Integrated phylogenetic and antigenic cartography analysis of 61 viruses using Bayesian multidimensional scaling (14). **(A)** Time-resolved phylogenetic tree of viruses circulating in England (black circles) and WHO reference viruses (red circles) with branches colored by inferred antigenic difference from A/California/07/2009 vaccine strain in antigenic map units. Study viruses are shown with text and vaccine virus names in bold. **(B)** Two-dimensional antigenic map showing antigenic (circles, colored by virus collection date) and phylogenetic relationships (lines) of viruses. Distances for antigenic dimensions are measured in antigenic units, where one unit represents a twofold dilution in heterologous hemagglutination inhibition titer. Study viruses (gray outline) and vaccine viruses (red outline) are highlighted.

We performed an integrated phylogenetic and antigenic cartography analysis (14) using hemagglutinin sequence data and HI titers for 61 (H1N1)pdm09 viruses collected between 2009 and 2016, comprising 53 viruses collected from England, 2 vaccine strains, and 6 other WHO reference viruses (Table S1 in Supplementary Material). Analysis of these genetic and antigenic data showed gradual genetic drift (Figure 4A) as well as gradual antigenic change (Figure 4B) of (H1N1)pdm09 viruses since 2009. The minimum antigenic distinction for when an influenza vaccine update is recommended is generally a difference between a vaccine strain and circulating strains of 2 antigenic map units, representing a fourfold drop in heterologous HI titer (15). Viruses circulating in England with at least 3 antigenic units (>8-fold drop in HI titer) difference from A/California/07/2009 only emerged from 2015 onward. A similar pattern was seen using multidimensional scaling (MDS) (Figure S1 in Supplementary Material). These recent viruses are antigenically similar to the updated H1N1 subtype component vaccine strain, A/Michigan/45/2015. Additionally, the recently circulating viruses include the emergence of one genetically distinct group of viruses, which are also genetically similar to the A/Michigan/45/2015 vaccine strain.

**Figure 5:**
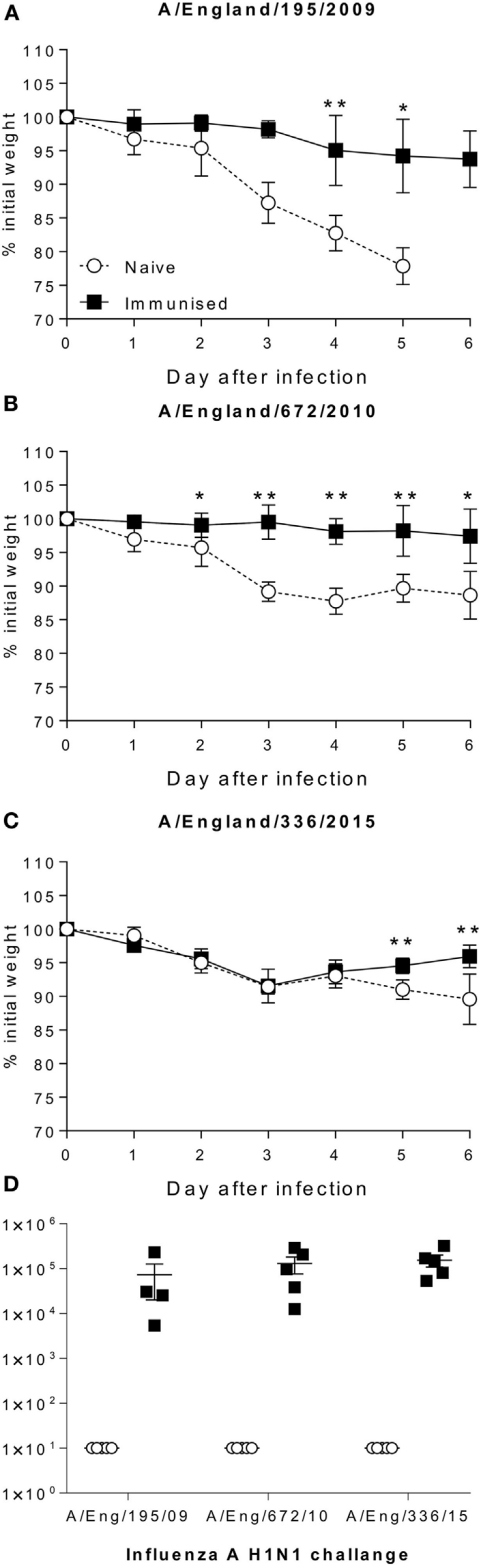
Antigenic drift in H1N1 strains is seen in mouse models. Mice were immunized with A/Cal/7/2009 (black squares) and challenged with A/England/195/2009 **(A)**, A/Eng/672/2010 **(B)**, A/England/336/2015 **(C)**, responses were compared to naïve animals (white circles). Antibody response to Cal/09 antigen prior to challenge **(D)**. Points mean of *n* = 4 mice ± SEM **(A–C)**, or individual animals **(D)**. *p < 0.05, **p < 0.01.

Since the H1 component of vaccine in use was unchanged from the initial wave of the pandemic, we wished to see whether the protection efficacy changed as the virus changed. Mice were immunized with 0.5 µg A/California/7/2009 antigens and then challenged either with a matched isolate from the initial wave of the pandemic in 2009 (A/England/195/2009), or drift isolates from 2010 (A/England/672/2010) or 2015 (A/England/336/2015). Immunized mice lost significantly less weight than control mice when infected with the 2009 (Figure 5A) or 2010 (Figure 5B) isolates. However mice infected with a 2015 isolate were not initially protected compared to the control animals, but they recovered slightly more rapidly than the unimmunized mice (Figure 5C). There was no significant difference in the antibody response to the immunizing antigen in the mice, suggesting that viral escape from this antigen drives the reduction in protection (Figure 5D).

## Discussion

In the current study, we have successfully developed mouse models of seasonal H1N1 influenza infection to test vaccine efficacy. Infection with current seasonal H1N1, but not H3N2 virus, led to disease in mice. Immunization of mice with a vaccine homologous to the challenge strain, protected against infection with the same strain. However, immunization of mice with A/California/7/2009 was not protective against challenge with an H1N1 strain from 2015. This may recapitulate the situation in humans where key changes in the clade 6B H1N1 viruses, not detected by classical serological tests, reduced protection in individuals exposed to an earlier H1N1 strain (16, 17).

Influenza virus infection in mice was characterized by a large percentage of the total body weight lost at the peak of disease, in some of the animals necessitating humane culling. There was not a noticeable change in appetite, so the most likely factor is increased effort in breathing driven by the very high levels of inflammation in the lungs. Previous studies have shown that blocking TNFα blocks reduces disease by reducing cell infiltration into the lower airways (18). We have recently demonstrated that cytokine release after influenza infection is localized to the lungs (19), suggesting that the inflammation is not systemic. It was notable that while the H1N1 and B viruses caused weight loss, nothing was seen after infection with a current H3N2 strain. The most likely reason for this is differences in receptor binding by the hemagglutinin molecules of the different viruses, though we do not have data on whether the H3N2 virus infection took in the mice. The current H1N1 subtype is able to bind both avian (α2,3-linked sialic acid) and human (α2,6-linked sialic acid), whereas the current H3N2 is more human adapted and only able to bind α2,6 (20, 21). Mice, like birds, only express the α2,3-linked receptor (22). Likewise influenza B can bind both α2,3 and α2,6 sialic acids (23). It should be noted that the isolation and propagation of B viruses used in this study may have introduced key mutations leading to the loss of glycosylation sites at 196 or 197 making them antigenically different from the circulating viruses.

Mice are widely used for preclinical vaccine studies. In the current study, we demonstrate that a very low dose of protein is protective against viral infection; this was especially the case when mice were immunized twice in a prime boost regime, where a 0.02 µg dose which was not protective after a single immunization was protective when given twice. One question is why they can be protected with such small doses of protein. One consideration is the dose to size ratio; the average mouse used in these studies is 25 g, the average human 62 kg a 2,500-fold scale up. The human formulation of flu vaccine normally contains 15 µg of each hemagglutinin, so an equivalent dose for a mouse would be 6 ng, which we saw was protective in the prime boost studies. Whether body mass is the best comparison is not clear, another consideration could be muscle size, with the human muscle approximately 400 times larger. The other consideration is that the immune response amplifies signal, especially when it is boosted with the same antigen; so potentially the consideration is not about the amount of protein rather how the cells involved in the response get to the site of immunization.

Putting vaccine dosing aside, a question is whether mice are easier to protect against infection than humans. It should be noted that the viruses used in these studies were not mouse adapted strains. In our study, we were using between 10^4^ and 10^6^ viruses; however in human deliberate challenge studies, a similar dose is used and gives varying levels of disease (24). Both mouse and human challenge studies may not reflect the situation in natural infection where the human infectious dose is believed to be between approximately 100 and 200 infectious virions (25), and a reanalysis of the same data set suggests that disease correlates with infectious dose (26). Studies in ferrets would suggest the dose is even lower, possibly between 3 and 10 virions (27). Since more virus is required to induce disease, it may be that less antibody is required to neutralize the virus and because of the smaller size of the mouse lung antibody may be more concentrated. While we didnot dissect the correlates of protection in the current study, in other studies the strongest correlate protection is IgG and we observed that immunization induced an influenza-specific IgG response. In addition to IgG, we have recently observed a role for IgA in both human (24) and mouse (28) challenge studies.

Vaccination with a protein antigen may restrict the specificity of the response to the immunizing antigen. This may especially be the case when the vaccine strain is unchanged over several rounds of immunization as was the case with the H1 antigen. Our data show a clear antigenic and immunogenic drift of the (H1N1)pdm09 virus from 2009 to 2016. Critically, a lack of protection against infection from the vaccine strain was observable with a virus isolated from the season before the vaccine strain was changed. Based on this, we would suggest that modeling in the mouse could be used to contribute to decisions about the efficacy of vaccination against the currently circulating strains of influenza H1N1.

## Materials and Methods

### Viruses

Seasonal influenza viruses (Table 2) were isolated by Public Health England (UK). The England strains of H1N1, A/England/195/2009, A/England/672/2010, and A/England/336/2015 were isolated in SIAT-MDCK cells (12). B viruses were expanded in eggs prior to being grown in Madin-Darby Canine Kidney (MDCK) cells. Prior to use in mice, viruses were propagated in MDCK cells, in serum-free DMEM supplemented with 1 µg/ml trypsin. The virus was harvested 3 days after inoculation and stored at -80°C. Viral titer was determined by plaque assay as described below.

**Table 2:** Influenza strains used in study.

Type	Surface subtype	Strain
A	H1N1	A/England/195/2009
A	H1N1	A/California/07/2009
A	H1N1	A/England/672/2010
A	H1N1	A/England/336/2015 (Clade 6B.1)
A	H1N1	A/Puerto Rico/8/1934
A	H3N2	A/England/691/2010
A	H3N2	A/X-31
B	Yam	B/Florida/04/06
B	Vic	B/Brisbane

### Mouse Immunization and Infection

6–10-week-old female CB6F1 mice were obtained from Harlan UK Ltd. (Horsham, UK) or from an internal breeding colony and kept in specific-pathogen-free conditions in accordance with the United Kingdom’s Home Office guidelines and all work was approved by the Animal Welfare and Ethical Review Board (AWERB) at Imperial College London. Studies followed the ARRIVE guidelines. Mice were immunized intramuscularly (i.m.) with purified surface antigens from influenza strain H1N1 A/California/7/2009 (GSK Vaccines, Siena, Italy) in 50 µl, either once (prime only) or twice (prime boost). For infections, mice were anesthetized using isoflurane and infected intranasally (i.n.) with 100 µl influenza virus or sterile PBS. Body surface temperature was taken from the xiphoid process using a hand-held infrared thermometer.

### Tissue and Cell Recovery and Isolation

Mice were culled using 100 µl intraperitoneal pentobarbitone (20 mg dose, Pentoject, Animalcare Ltd., UK) and tissues collected as previously described (29). Blood was collected from femoral veins and sera isolated after clotting by centrifugation. Lungs were removed and homogenized by passage through 100-µm cell strainers, then centrifuged at 200 × *g* for 5 min. Supernatants were removed and the cell pellet treated with red blood cell lysis buffer (ACK; 0.15 M ammonium chloride, 1 M potassium hydrogen carbonate, and 0.01 mM EDTA, pH 7.2) before centrifugation at 200 × *g* for 5 min. The remaining cells were resuspended in RPMI 1640 medium with 10% fetal calf serum, and viable cell numbers determined by trypan blue exclusion.

### Histology

Upper and lower regions of paraformaldehyde-fixed left lung lobes were processed and embedded in paraffin. Sections of 3 µm were stained with hematoxylin and eosin and the entire section was scanned at ×20 magnification so that the area with the greatest inflammation could be assigned the inflammation score. The degree of airway inflammation was assessed in a blinded manner using a modified system described previously (30). Briefly, the degree of inflammation in the peribronchiolar, perivascular, and interstitial regions of both the upper and lower airways was assessed. A value of 0 (none), 1 (minimal), 2 (mild), 3 (moderate), or 4 (severe) was given to each histological site and the sum of these scores was used as the total upper/lower respiratory inflammation score.

### Influenza Viral Load

#### Viral RNA Quantification

Viral load *in vivo* was assessed by Trizol extraction of RNA from frozen lung tissue disrupted in a TissueLyzer (Qiagen, Manchester, UK). RNA was converted into cDNA and quantitative RT-PCR was carried out using bulk viral RNA, for the influenza M gene and mRNA using 0.1 µM forward primer (5′-AAGACAAGACCAATYCTGTCACCTCT-3′), 0.1 µM reverse primer (5′-TCTACGYTGCAGTCCYCGCT-3′) and 0.2 µM probe (5′-FAM-TYACGCTCACCGTGCCCAGTG-TAMRA-3′) on a Stratagene Mx3005p (Agilent technologies, Santa Clara, CA, USA). M-specific RNA copy number was determined using an influenza M gene standard plasmid.

#### Plaque Assays

Plaque assays were performed using a modified protocol previously described (31). Briefly, confluent monolayers of MDCK cells in 12-well plates were inoculated with 200 µl of viral or sample dilutions and incubated for 1 h. The inoculum was removed then the cells were overlaid with 0.6% agarose (Oxoid) in MEM including 1 µg/ml trypsin and incubated at 37°C with 5% CO_2_. After 3 days, the agarose was removed and the cells stained with crystal violet dissolved in methanol and water.

#### Flow Cytometry

Live cells were suspended in Fc block (Anti-CD16/32, BD) in PBS-1% BSA and stained with surface antibodies: CD3-FITC (BD, Oxford UK), CD4-APC (BD), CD8-APC Alexa75 (Invitrogen, Paisley, UK), and NK1.1 PerCP-Cy5.5 (BD, Oxford UK). Analysis was performed on an LSRFortessa flow cytometer (BD). FMO controls were used for surface stains.

### Semi-Quantitative Antigen-Specific ELISA

Antibodies specific to influenza H1N1 were measured using a standardized ELISA (32). IgG responses were measured in sera. MaxiSorp 96-well plates (Nunc) were coated with 1 µg/ml surface proteins or a combination of anti-murine lambda and kappa light chain specific antibodies (AbDSerotec, Oxford, UK) and incubated overnight at 4°C. Plates were blocked with 1% BSA in PBS. Bound IgG was detected using HRP-conjugated goat anti-mouse IgG (AbD Serotec). A dilution series of recombinant murine IgG was used as a standard to quantify specific antibodies. TMB with H_2_SO_4_ as stop solution was used to detect the response and optical densities read at 450 nm.

### Integrated Analysis of Antigenic and Genetic Evolution

A Bayesian multidimensional scaling (BMDS) model (14) of antigenic cartography (15) was implemented in BEAST v1.8.4 (33) to jointly infer antigenic and phylogenetic relationships, as previously described (14, 34). Briefly, an antigenic dataset composed of available HA gene sequences and corresponding HI measurements was assembled for 61 H1N1pdm09 viruses collected between 2009 and 2016: 53 viruses circulating in England, 2 vaccine strains, and 6 WHO reference viruses. Viruses were originally isolated from clinical specimens either by WHO NICs or by the WHO Collaborating Center and the corresponding HI measurements were obtained either from the Francis Crick Institute Influenza Interim Reports or were kindly provided by John McCauley. HA sequences were downloaded from either the Influenza Research Database (35) or the EpiFlu database (36). A phylogenetic tree of the HA sequences was estimated using BEAST (33) which incorporated the HKY substitution model, a coalescent model with constant effective population size and a strict molecular clock. Markov chain Monte Carlo (MCMC) was run for 15 million steps and trees were logged every 1,500 steps, with a burn-in of 5 million steps, resulting in 10,000 trees. This posterior set of 10,000 trees was used with the HI data to implement the full BMDS model infer virus and serum locations in two antigenic dimensions, as well as virus avidities, serum potencies, MDS precision, and virus and serum location precisions in BEAST. MCMC chains were run for 500 million states with sampling every 200,000 states with 10% burn-in, and run convergence was checked in Tracer v1.6 (http://tree.bio.ed.ac.uk/software/tracer/). A maximum clade credibility tree was summarized in TreeAnnotator v1.8.4 (33) and visualized using FigTree v1.4.3 (http://tree.bio.ed.ac.uk/software/figtree/). Antigenic map plots were generated using custom Python scripts with the matplotlib library (37).

### Statistical Analysis

Calculations as described in figure legends were performed using Prism 6 (GraphPad Software Inc., La Jolla, CA, USA).

## Ethics Statement

Work was performed in accordance with the United Kingdom’s Home Office guidelines and all work was approved by the Animal Welfare and Ethical Review Board (AWERB) at Imperial College London. Studies followed the ARRIVE guidelines.

## Author Contributions

HG, JM, and EK performed the experimental studies; PL and PK performed the data analysis of flu strain drift; JM provided HI data and analysis; JE, CT, RE, LP, and WB provided and grew influenza strains, JT designed the studies and wrote the paper.

## Conflict of Interest Statement

The authors declare that the research was conducted in the absence of any commercial or financial relationships that could be construed as a potential conflict of interest.
