# Supplementary figures and images for: Mouse Models of Influenza Infection with Circulating Strains to Test Seasonal Vaccine Efficacy
Source: Front Immunol. 2018 Jan 31;9:126. doi: 10.3389/fimmu.2018.00126 (PMC5797846; doi:10.3389/fimmu.2018.00126)

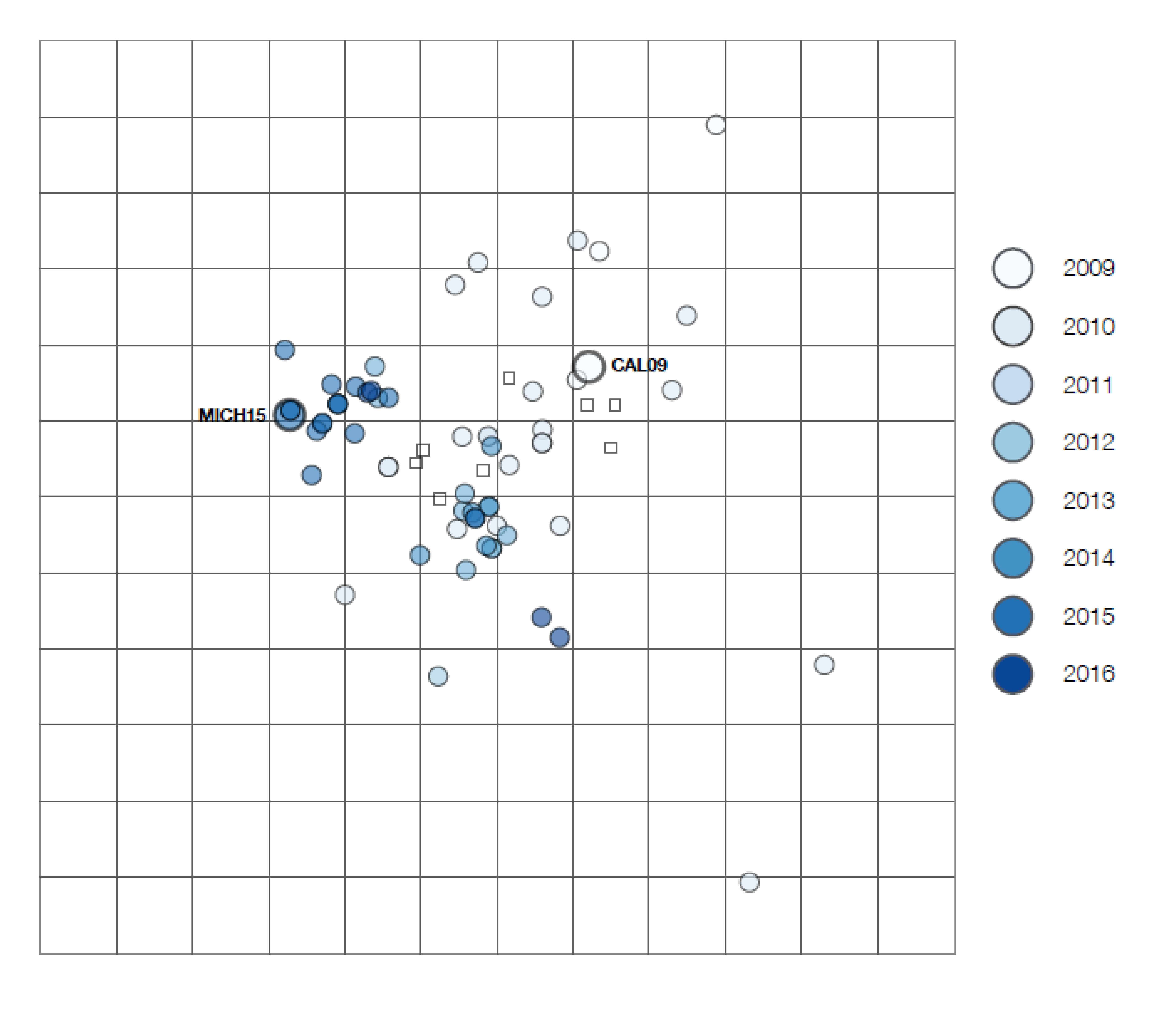

Supplement: Supplementary file 2 [file Image_1.TIF]
